# FGFRL1 affects chemoresistance of small‐cell lung cancer by modulating the PI3K/Akt pathway via ENO1

**DOI:** 10.1111/jcmm.14763

**Published:** 2020-01-19

**Authors:** Rui Chen, Deyu Li, Meng Zheng, Bin Chen, Ting Wei, Yu Wang, Man Li, Weimei Huang, Qin Tong, Qi Wang, Yaru Zhu, Wei Fang, Linlang Guo, Shun Fang

**Affiliations:** ^1^ Department of Pathology Zhujiang Hospital Southern Medical University Guangzhou China; ^2^ Department of Oncology Jiujiang First People's Hospital Jiujiang China; ^3^ Department of Medical Oncology Provincial Clinical College Fujian Provincial Hospital Fujian Medical University Fuzhou China; ^4^ Department of Hepatic Surgery The First Affiliated Hospital Sun Yat‐sen University Guangzhou China; ^5^ Department of Oncology Zhujiang Hospital Southern Medical University Guangzhou China; ^6^ Department of Cardiothoracic Surgery Zhujiang Hospital Southern Medical University Guangzhou China; ^7^ Department of General Surgery Zhujiang Hospital Southern Medical University Guangzhou China

**Keywords:** chemoresistance, ENO1, FGFRL1, PI3K/Akt pathway, small‐cell lung cancer

## Abstract

Fibroblast growth factor receptor‐like 1 (FGFRL1), a member of the FGFR family, has been demonstrated to play important roles in various cancers. However, the role of FGFRL1 in small‐cell lung cancer (SCLC) remains unclear. Our study aimed to investigate the role of FGFRL1 in chemoresistance of SCLC and elucidate the possible molecular mechanism. We found that FGFRL1 levels are significantly up‐regulated in multidrug‐resistant SCLC cells (H69AR and H446DDP) compared with the sensitive parental cells (H69 and H446). In addition, clinical samples showed that FGFRL1 was overexpressed in SCLC tissues, and high FGFRL1 expression was associated with the clinical stage, chemotherapy response and survival time of SCLC patients. Knockdown of FGFRL1 in chemoresistant SCLC cells increased chemosensitivity by increasing cell apoptosis and cell cycle arrest, whereas overexpression of FGFRL1 in chemosensitive SCLC cells produced the opposite results. Mechanistic investigations showed that FGFRL1 interacts with ENO1, and FGFRL1 was found to regulate the expression of ENO1 and its downstream signalling pathway (the PI3K/Akt pathway) in SCLC cells. In brief, our study demonstrated that FGFRL1 modulates chemoresistance of SCLC by regulating the ENO1‐PI3K/Akt pathway. FGFRL1 may be a predictor and a potential therapeutic target for chemoresistance in SCLC.

## 1 INTRODUCTION

Lung carcinoma is the primary cause of cancer mortality in globally, with millions of new cases diagnosed annually.^1^ SCLC is a neuroendocrine malignancy with poor prognosis, accounting for approximately 15% of lung cancer cases.^2^ SCLC is generally classified into limited disease and extensive disease. Platinum in combination with etoposide is the first‐line treatment for SCLC, and the majority of patients are susceptible to initial chemotherapy. However, most SCLC patients rapidly develop chemoresistance, and the 2‐year survival rate is less than 5%.^3^, ^4^ Thus, the study of chemoresistance in SCLC is vital.

FGFRL1 is known as a member of the fibroblast growth factor receptor (FGFR) family. The FGFR family is generally composed of three extracellular ig‐like domains, a transmembrane helical region and an intracellular tyrosine kinase domain. However, FGFRL1 lacks the classic tyrosine kinase regions and contains a peculiar histone‐rich region instead.^5^ Therefore, FGFRL1 is widely considered to negatively regulate the FGF signalling pathway by combining extracellular ligand to prevent its interaction with typical receptors. Several studies have generated data supporting this hypothesis,^6^, ^7^ whereas others have found conflicting results.^8^, ^9^, ^10^ Gerber et al^9^ demonstrated that FGFRL1 may be a positive regulator of the FGF signalling pathway rather than a decoy receptor during renal development. Furthermore, FGFRL1 enhances the ERK1/2 signalling pathway by interacting with SHP1 in pancreatic beta cells.^10^ Recently, increasing evidence has shown that FGFRL1 plays a key role in many cancers, and overexpression of FGFRL1 has been associated with proliferation and metastasis of prostate and gastric cancer cells.^11^, ^12^ In addition, some studies have demonstrated that micoRNAs can inhibit the growth and metastasis of tumours by targeting FGFRL1.^12^, ^13^, ^14^ FGFRL1 can also enhance the malignant phenotype of ovarian cancer cells by activating Hedgehog signalling,^15^ and FGFRL1 is an important prognostic factor in patients with oesophageal cancer.^16^ However, the study of FGFRL1 in SCLC has rarely been reported.

Enolase (ENO) proteins are glycolytic enzymes that catalyse the dehydration of 2‐phospho‐D‐glycerate (2‐pg) to phosphoenolpyruvate (PEP) during glycolysis. In mammals, there are three isoforms of ENO: alpha‐, beta‐ and gamma‐enolase, and alpha‐enolase (ENO1) is widely expressed in most tissues.^17^ In tumours, ENO1 is overexpressed and activated, which enhances the glycolytic process.^18^ Previous studies have indicated that ENO1 promotes malignant biological behaviour of tumour cells by activating glycolysis and the PI3K/Akt pathway in non–small‐cell lung cancer and glioma cells.^19^, ^20^, ^21^ In addition, ENO1 can regulate the malignant biological functions of pulmonary artery smooth muscle cells through the AMPK/Akt signalling pathway.^22^ More importantly, some lncRNAs and proteins have been reported to interact with ENO1 to regulate its expression or activity in tumours.^19^, ^23^, ^24^

This study is the first to show that FGFRL1 is involved in SCLC. We demonstrated that FGFRL1 was up‐regulated in multidrug‐resistant SCLC cells compared with the parental sensitive cells. Functionally, FGFRL1 promotes chemoresistance of SCLC in vivo and in vitro. Mechanistically, FGFRL1 interacts with and increases the expression of ENO1 to further activate the PI3K/Akt pathway. Taken together, these studies reveal a new mechanism of chemoresistance in SCLC.

## 2 MATERIALS AND METHODS

### 2.1 Cell culture and reagents

The human SCLC cell lines H69, H446 and H69AR were purchased from American Type Culture Collection (ATCC, USA). H446DDP was constructed by exposing H446 cells to cisplatin for 6 months in our laboratory. The IC50 values of these four cells are shown in Figure S1A. H69AR and H446DDP were maintained in drug‐free medium for at least 2 weeks before experiments. All cells were cultured in RPMI‐1640 medium (Hyclone) containing 10% foetal bovine serum (Gibco) and antibiotics (100 mg/mL penicillin and 100 mg/mL streptomycin).

ENOblock and LY294002 were purchased from Selleck Chemicals. To inhibit the function of ENO1 or the activities of the PI3K/Akt pathway, cells were treated with 10 μmol/L ENOblock or 12 μmol/L LY294002 for 48 hours. The following antibodies were used: anti‐FGFRL1, anti‐HuR and anti‐ENO1 were purchased from Abcam; anti‐BAX, anti‐BCL2 and anti‐p‐Akt 427 were from Proteintech; anti‐PARP, anti‐p‐PI3K and anti‐p‐Akt 308 were from Affinity; anti‐T‐Akt and anti‐T‐PI3K were from Wanlei; and anti‐GAPDH was from Bioworld.

### 2.2 Patients and tissue samples

In this study, a total of 36 formalin‐fixed paraffin‐embedded (FFPE) tissues were obtained from SCLC patients who underwent bronchofiberscopy or biopsy between the period of January 2013 and December 2016 and received nursing care and follow‐up in Fujian Provincial Hospital (Fujian, China). Non‐cancerous lung tissues including bronchiectasis and pulmonary bulla were all from benign lung diseases. A chemotherapy response was categorized as ‘chemotherapy sensitivity’ (partial response or complete response) and ‘chemotherapy refractory’ (progressive or stable disease) based on the Response Evaluation Criteria in Solid Tumors (RECIST [edition 1.1]). Our study was approved by the hospital's Protection of Human Subjects Committee, and informed consent was obtained from all patients.

### 2.3 RNA isolation and real‐time qRT‐PCR

Total RNA was obtained from FFPE tissues and cells using the RNeasy FFPE Kit (Qiagen) and TRIzol reagent (Invitrogen), based on the manufacturer's instructions. A NanoDrop 2000 (Thermo) was used to measure the RNA concentrations. Then, qRT‐PCR was performed with an ABI Illumina instrument (Foster, USA) using SYBR Green (Tiangen). The relative mRNA expression levels were obtained by the 2^−ΔΔCT^ method. All primers are shown in Table S1.

### 2.4 Western blot analysis

Total protein was obtained from cells and tissues with RIPA lysis buffer (Biyuntian). The protein concentrations were measured using a BCA Protein Quantitation Kit (Cwbio). Equivalent amounts of protein lysates were separated by 10% SDS‐polyacrylamide gel electrophoresis and then transferred to a PVDF membrane. Membrane was blocked with 5% bovine serum albumin (BSA) for 2 hours and then incubated with specific primary antibodies overnight at 4°C. After three washes with 1 × TBST, the membranes were incubated with HRP‐conjugated anti‐rabbit or antimouse secondary antibody (EarthOx). Finally, the proteins were detected by chemiluminescence (ECL).

### 2.5 Transfection

According to the manufacturer's instructions, we transiently transfected cells with siRNAs against FGFRL1 and ENO1 (GenePharma) using Lipofectamine 3000 (Thermo Scientific) and Opti‐MEM (Invitrogen). The sequences targeting FGFRL1 and ENO1 are listed in Table S2.

For stable transfection, lentiviral particles encoding shFGFRL1, shNC, FGFRL1‐GFP or NC‐GFP (GenePharma) were transfected into SCLC cells. After infection for 48 hours, cells were selected with 2.0 μg/mL puromycin (Solarbio). Transfection efficiency was validated by qRT‐PCR and Western blot.

### 2.6 Drug resistance assay (CCK8 assay)

After confirmation of transient or stable transfection, the cells were incubated with the chemotherapy drugs adriamycin (ADM), cisplatin (CDDP) or etoposide (VP‐16, Jiangsu) for 24 hours. After treatment with 10 μL CCK‐8 reagent (Dojindo) for 3 hours, absorbance of the cells was detected at 450 nm. The IC50 of each chemotherapeutic drug was calculated according to the OD value.

### 2.7 Tumour xenograft experiments

Female BALB/c nude mice aged 4‐5 weeks were purchased from the Experimental Animal Center of Southern Medical University (Guangzhou, China). The experiments were approved according to the institutional guidelines of Guangdong Province and the Use Committee for Animal Care and were performed based on guidelines of the Association for Assessment and Accreditation of Laboratory Animal Care International. Stably transfected cells were acquired and resuspended in PBS at a concentration of 1 × 10^7^ cells per 0.1 mL. Twenty‐four mice were randomly distributed into four treatment groups of H69AR cells (shFGFRL1 or shNC) treated with chemotherapy or PBS, and H69 cells (FGFRL1‐GFP or NC‐GFP) treated with chemotherapy or PBS. Each mouse was injected with SCLC cells subcutaneously in the flanks. After 1 week, the mice were intraperitoneally injected with PBS or chemotherapy drugs (CDDP 3 mg/kg and VP‐16 2 mg/kg) once every 4 days. Subcutaneous tumours were measured every 4 days, and mice were killed after 4 weeks. Subsequently, the tumours were photographed and analysed via qRT‐PCR and Western blot.

### 2.8 Flow cytometric analysis

Transfected cells were treated with chemotherapy drugs (ADM, CDDP and VP‐16) for 24 hours and then collected for further experiments. The dosage of chemotherapy drugs is equivalent to 1/2 of normal cell IC50. Apoptosis was evaluated using Annexin V450/eff660 APC (eBioscience) based on the manufacturer's protocol. For the cell cycle assay, the cells were fixed for 4 hours with 75% ethanol and then stained with propidium iodide (Sigma). All samples were analysed by a BD FACS Verse flow cytometer.

### 2.9 Co‐immunoprecipitation assay

Following the manufacturer's protocol, co‐immunoprecipitation (Co‐IP) assays were conducted using the Thermo Scientific Pierce Co‐IP Kit (Thermo Scientific). The immunoprecipitated proteins were analysed by Western blot.

### 2.10 Mass spectrometry

The antibody complexes obtained by Co‐IP were analysed by LC‐MS/MS, and accurate high‐resolution mass data were obtained using a Q Exactive Orbitrap mass spectrometer (Thermo Scientific).

### 2.11 Immunofluorescence staining

A small number of cells were seeded on coverslips. After 12 hours, cells were fixed with 4% paraformaldehyde and permeabilized with 0.3% Triton X‐100. Cells were blocked for 2 hours with 5% BSA and then treated with specific antibody overnight at 4°C. After three washed with PBS, the cells were incubated with specific secondary antibody (Invitrogen) in the dark for 1 hour. Finally, cell nuclei were stained with DAPI, and images were obtained by fluorescence microscopy.

### 2.12 Statistical analysis

SPSS (SPSS, Chicago, USA) and GraphPad Prism (GraphPad Software Inc) were used for the statistical analysis. The results are represented as the mean ± standard deviation (SD). Statistical differences were analysed by independent‐sample *t* tests or one‐way analysis of variance. The associations between FGFRL1 expression and clinical features were analysed by chi‐square test or Fisher's exact test. Survival curves were assessed by Kaplan‐Meier analysis. *P < *.05 was considered statistically significant.

## 3 RESULTS

### 3.1 FGFRL1 expression is increased in chemoresistant SCLC cell lines and SCLC tissues

**Figure 1:**
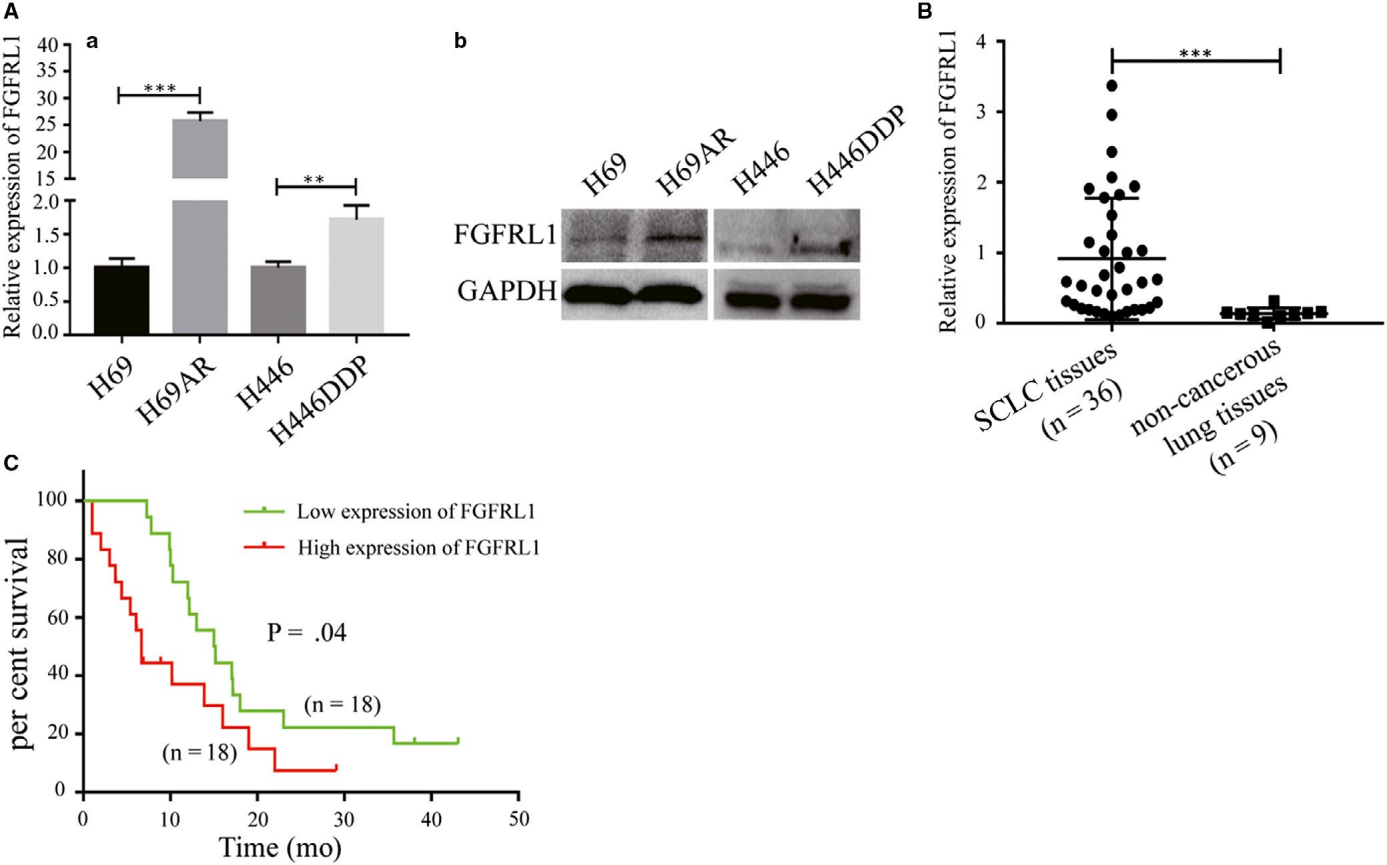
FGFRL1 expression is increased in chemoresistant SCLC cell lines and SCLC tissues. A, qRT‐PCR (a) and Western blot (b) analysis of FGFRL1 expression in chemoresistant cells (H69AR and H446DDP) and their parental cells (H69 and H446). B, The expression of FGFRL1 in SCLC tissues (n = 36) and non‐cancerous lung tissues (n = 9). C, Kaplan‐Meier analysis of overall survival of 36 patients with SCLC based on FGFRL1 expression. **P < .01; ***P < .001

The genome‐wide gene expression analysis showed a 28‐fold up‐regulation of FGFRL1 in multidrug‐resistant SCLC cells (H69AR) compared with parental cells (H69) (Table S3). This result was verified at mRNA and protein levels in two pairs of chemoresistant SCLC cell lines (Figure 1A).

To further investigate the clinicopathological features of FGFRL1, FGFRL1 expression was measured by qRT‐PCR in 36 SCLC tissue samples and 9 non‐cancerous lung tissue samples. The results showed that the expression of FGFRL1 in SCLC tissues was higher than that in non‐cancerous lung tissues (Figure 1B; cell levels also confirm the conclusion Figure S1B). We found that high expression of FGFRL1 was associated with poor patient survival by Kaplan‐Meier survival analysis (Figure 1C), and Table 1 shows the relationship between FGFRL1 expression and clinical data of SCLC patients. The result suggests that high expression of FGFRL1 is correlation to increased clinical stage, clinical chemotherapy resistance and smoking history in SCLC. However, there was no marked association between FGFRL1 expression and age or gender. In brief, these results indicate that FGFRL1 is highly expressed in SCLC‐resistant cells and SCLC tissues, and its high expression is associated with stage and survival of SCLC patients.

**Table 1:** The relationship between FGFRL1 expression and clinical parameters in 36 SCLC patients

Characteristics	Total	FGFRL1 expression		*P*‐value
		Low expression	High expression	
Gender				
Male	32	15	17	.603
Female	4	3	1	
Age (y)				
≥60 y	20	11	9	.502
<60 y	16	7	9	
Smoking history				
Yes	24	9	15	.034
No	12	9	3	
Disease stage				
LD	19	13	6	.019
ED	17	5	12	
Response				
Sensitive	20	13	7	.044
Refractory	16	5	11	

### 3.2 FGFRL1 expression is correlated with chemoresistance of SCLC in vitro and in vivo

**Figure 2:**
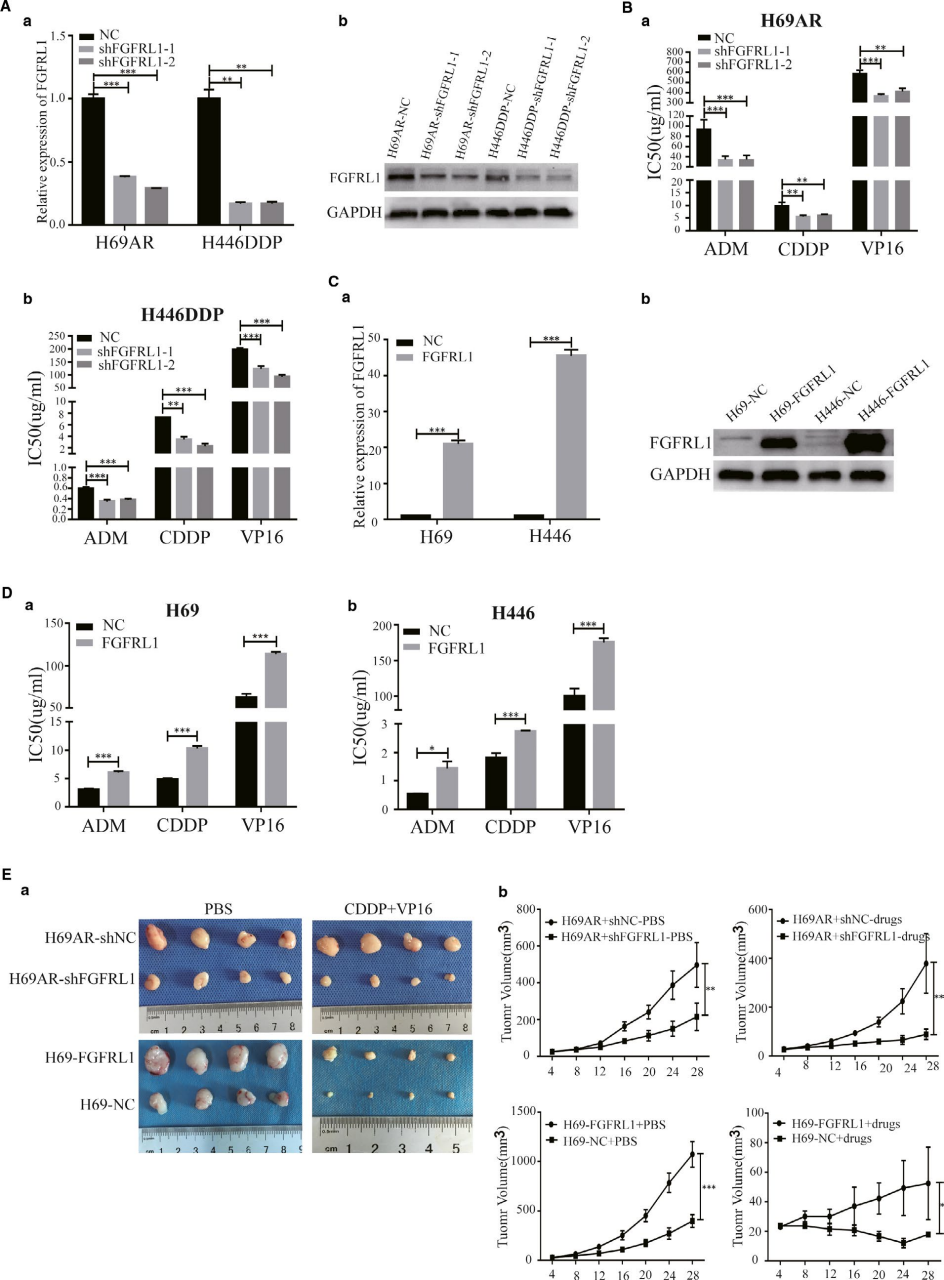
FGFRL1 expression was correlated with chemoresistance of SCLC in vitro and in vivo. A, Inhibition of FGFRL1 by transfection of FGFRL1 shRNA in H69AR and H446DDP cells. B, FGFRL1–down‐regulated cells were exposed to chemotherapy drugs, and IC50 values were assessed by CCK8 assays. C, Overexpression of FGFRL1 by transfection of pcDNA3.1‐FGFRL1 in H69 and H446 cells. D, IC50 values were measured by CCK8 assays when FGFRL1‐overexpressing cells were exposed to chemotherapy drugs. E, Tumours from mice in each group and the growth curve showing all tumour volumes. *P < .05; **P < .01; ***P < .001

In order to assess whether FGFRL1 was functionally involved in the chemoresistance of SCLC, we designed four different FGFRL1 siRNAs to transfect H69AR cells. qRT‐PCR and Western blot were performed at 48 hours post‐transfection and showed that siFGFRL1‐1 and siFGFRL1‐2 had higher knockdown efficiency than siFGFRL1‐3 and siFGFRL1‐4 (Figure S1C). Therefore, we chose siFGFRL1‐1 and siFGFRL1‐2 for the subsequent experiments. We also established stable FGFRL1 knockdown in H69AR and H446DDP cell lines by retrovirus infection (Figure 2A). CCK8 assays were conducted to evaluate the chemosensitivity of SCLC cells to various drugs (ADM, CDDP and VP‐16). The results showed that the IC50 values were significantly decreased after knockdown of FGFRL1 in H69AR and H446DDP cells (Figure 2B).

To complement these results, we overexpressed FGFRL1 in parental sensitive H69 and H446 SCLC cells. qRT‐PCR and Western blot analysis showed that FGFRL1 expression remarkable increased in H69‐FGFRL1 and H446‐FGFRL1 cells (Figure 2C). As expected, overexpression of FGFRL1 resulted in chemoresistance. The IC50 value of FGFRL1‐transfected cells increased significantly with chemotherapy drug treatment compared with the empty vector control (Figure 2D).

To investigate whether FGFRL1 confers chemoresistance of SCLC in vivo, we subcutaneously transplanted H69AR or H69 cells with altered FGFRL1 expression into nude mice. FGFRL1 knockdown significantly decreased the tumour volumes after treatment with PBS or drugs; in contrast, the tumour volume of the FGFRL1 overexpression group was significantly increased compared with the corresponding control group (Figure 2E). Transfection efficiency of FGFRL1 in tumour xenografts was detected by qRT‐PCR and Western blot (Figure S1D). These results suggest that FGFRL1 can affect the chemoresistance of SCLC cells in vitro and in vivo.

### 3.3 FGFRL1 induces chemoresistance of SCLC mainly by decreasing drug‐induced apoptosis and cell cycle arrest

**Figure 3:**
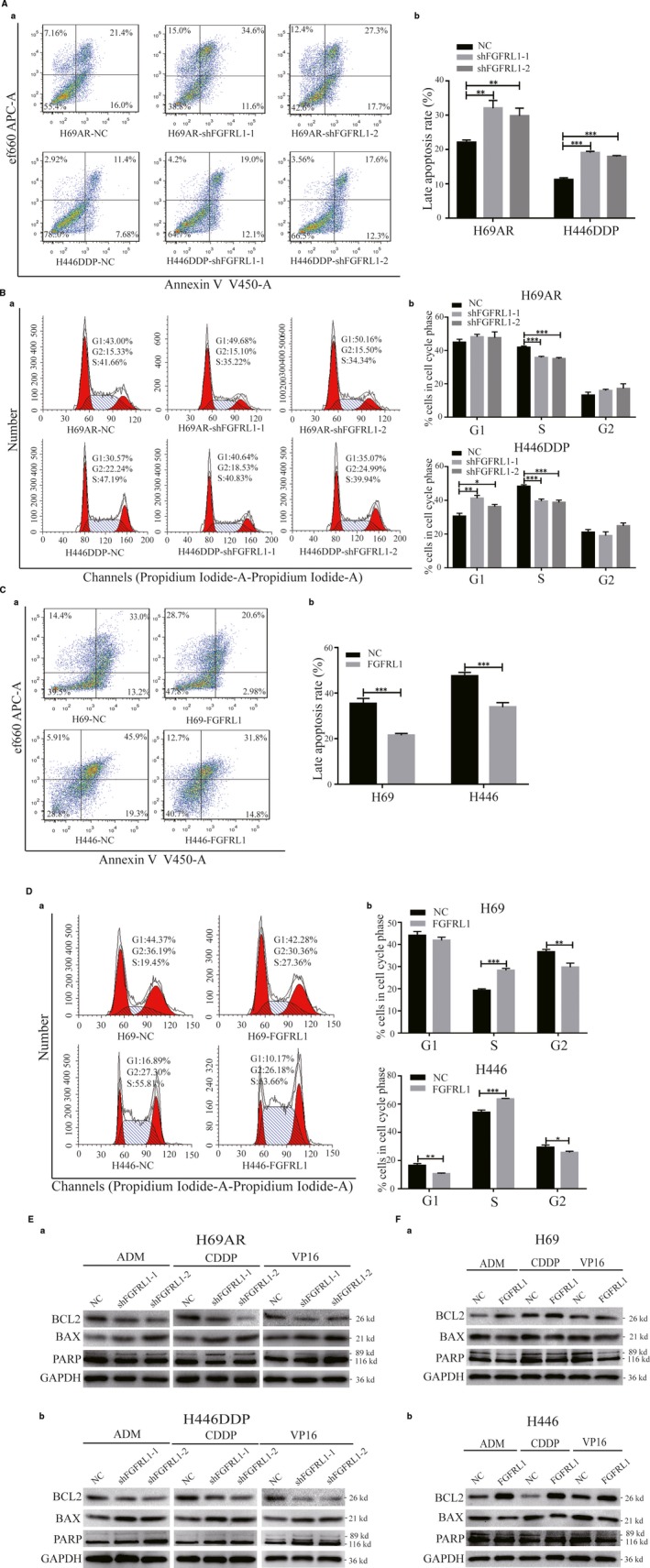
FGFRL1 induces chemoresistance of SCLC mainly by decreasing drug‐induced apoptosis and cell cycle arrest. A, B, Cell apoptosis and cell cycle arrest were evaluated by flow cytometric analysis in FGFRL1–down‐regulated SCLC cells after ADM exposure. C, D, Flow cytometric analysis of cell apoptosis and cell cycle arrest induced by ADM in FGFRL1‐overexpressing SCLC cells. E, F, Apoptosis‐related proteins were measured by Western blot following anticancer drug exposure in SCLC cells with down‐regulated or up‐regulated FGFRL1 expression. **P < .01; ***P < .001

To explore the possible mechanism of FGFRL1 resistance in SCLC, we assessed the effect of FGFRL1 on apoptosis and the cell cycle of cell exposure to chemotherapeutic drugs. Upon treatment of the cells with anticancer drugs, down‐regulation of FGFRL1 in H69AR and H446DDP cells increased cell apoptosis (Figure 3A) and cell cycle arrest (Figure 3B) according to flow cytometry analysis. In contrast, FGFRL1 overexpression in H69 and H446 cells decreased cell apoptosis (Figure 3C) and cell cycle arrest (Figure 3D; data on CDDP and VP16 are given in Figures S2 and S3). In addition, the ratio of Bax protein to both Bcl‐2 and cleaved PARP increased in FGFRL1‐knockdown cells after treatment with ADM, CDDP or VP16 (Figure 3E), whereas FGFRL1 overexpression in H69 and H446 cells produced the opposite result (Figure 3F). These results suggest that FGFRL1 may influence the chemoresistance of SCLC by impairing apoptosis and cell cycle arrest.

### 3.4 FGFRL1 interacts with ENO1 in SCLC cells

**Figure 4:**
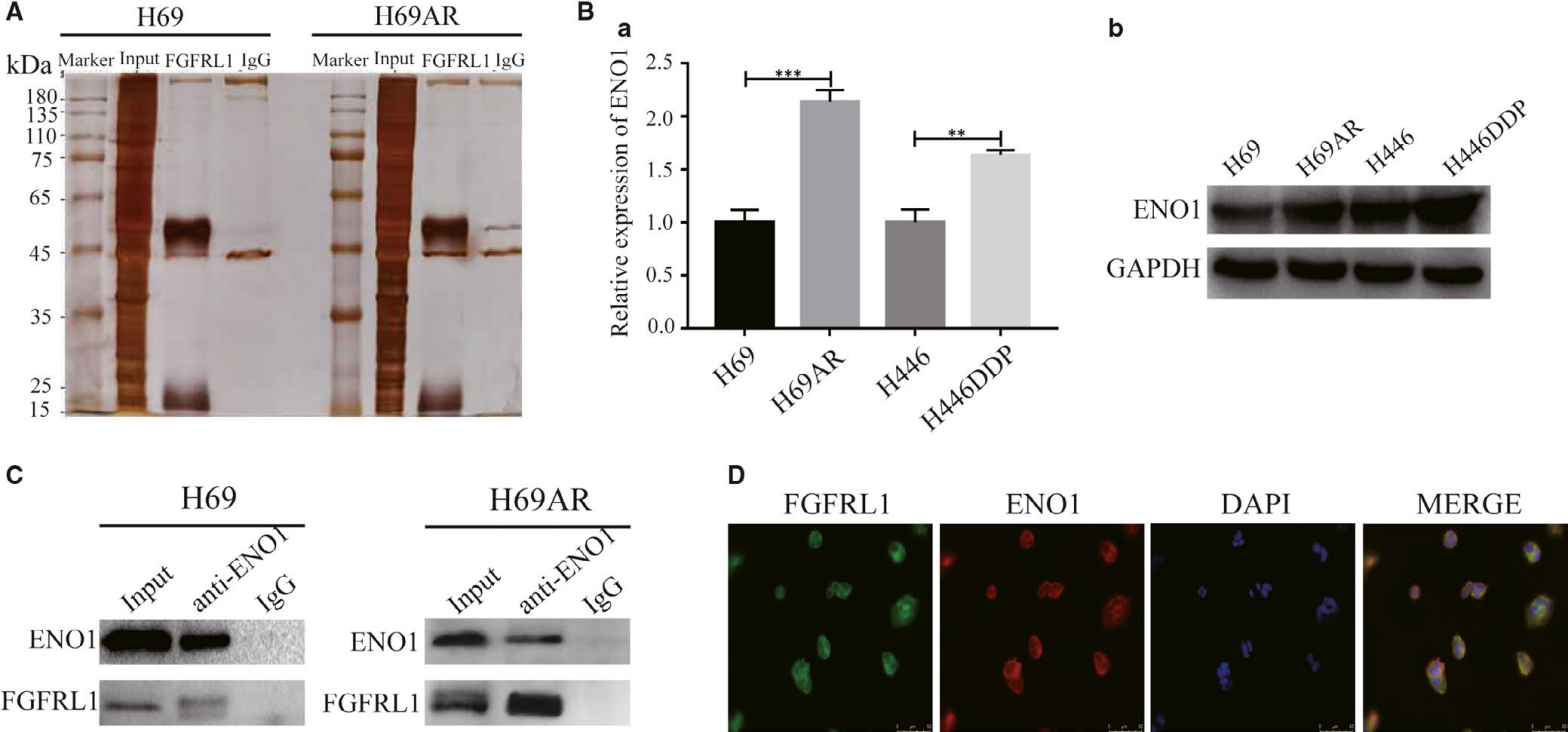
FGFRL1 interacts with ENO1 in SCLC cells. A, SDS‐PAGE showing proteins obtained by large‐scale Co‐IP. B, qRT‐PCR and Western blot analysis of ENO1 expression in SCLC cells. C, Co‐IP assays were conducted with specific ENO1 antibody in H69 and H69AR cells. D, Co‐localization of FGFRL1 and ENO1 was tested by immunofluorescence assay in H69AR cells. **P < .01; ***P < .001

In an effort to elucidate how FGFRL1 affects chemoresistance of SCLC, protein‐protein interaction mechanisms were investigated. Proteins potentially interacting with FGFRL1 were identified by immunoprecipitation‐mass spectrometry in H69 and H69AR cells (Figure 4A and Tables 2, 3). We screened a potential partner protein, ENO1, which showed high coverage with FGFRL1 and elevated expression in the chemoresistant SCLC cells (Figure 4B). To verify the immunoprecipitation‐mass spectrometry result, we performed a Co‐IP assay using an ENO1‐specific antibody. The result confirmed that ENO1 physically interacted with FGFRL1 in both H69 and H69AR cells (Figure 4C). Moreover, subcellular co‐localization of FGFRL1 and ENO1 was observed by immunofluorescence assay in H69AR cells (Figure 4D). Together, these results indicate that FGFRL1 interacts with ENO1 in SCLC cells.

**Table 2:** Potential interacting protein partners of FGFRL1 in H69 cells

Accession	Gene	Coverage	Peptides	Avg. mass	Description
Q9BYV8	CEP41	51	18	41 368	Centrosomal protein of 41 kD
Q9BQE3	TBA1C	35	11	49 895	Tubulin alpha‐1C chain
P68104	EF1A1	24	9	50 141	Elongation factor 1‐alpha 1
Q9BY77	PDIP3	15	4	46 089	Polymerase delta‐interacting protein 3
P06733	ENOA	16	4	47 169	Alpha‐enolase
P31943	HNRH1	10	3	49 229	Heterogeneous nuclear ribonucleoprotein

**Table 3:** Potential interacting protein partners of FGFRL1 in H69AR cells

Accession	Gene	Coverage	Peptides	Avg. mass	Description
Q9BYV8	CEP41	45	13	41 368	Centrosomal protein of 41 kD
Q9BQE3	TBA1C	31	10	49 895	Tubulin alpha‐1C chain
P68104	EF1A1	18	6	50 141	Elongation factor 1‐alpha 1
P06733	ENOA	13	3	47 169	Alpha‐enolase
Q9BY77	PDIP3	6	2	46 089	Polymerase delta‐interacting protein 3
P31943	HNRH1	8	2	49 229	Heterogeneous nuclear ribonucleoprotein
P26641	EF1G	8	3	50 119	Elongation factor 1‐gamma

### 3.5 FGFRL1 promotes chemoresistance of SCLC through ENO1

**Figure 5:**
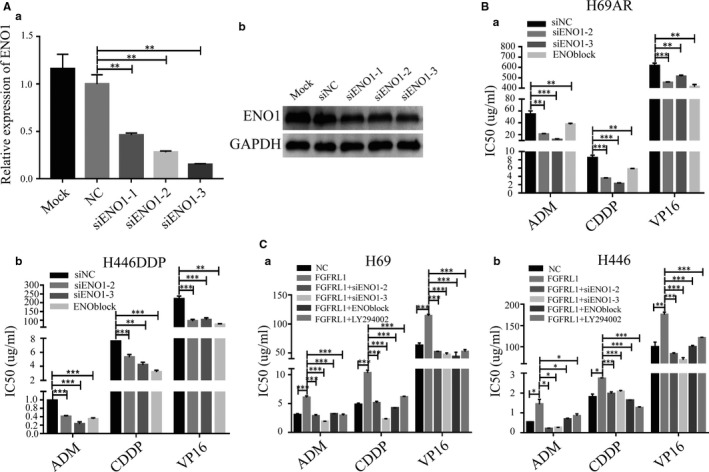
FGFRL1 promotes chemoresistance of SCLC through ENO1. A, Inhibition of ENO1 by transfection of ENO1 siRNA in H69AR cells. B, ENO1–down‐regulated or ENO1‐inhibited cells were exposed to chemotherapy drugs, and IC50 values were measured by CCK8 assays. C, IC50 values were tested by CCK8 assays after transfection of chemosensitive cells with negative control vector, pcDNA3.1‐FGFRL1, pcDNA3.1‐FGFRL1 + siENO1‐2, pcDNA3.1‐FGFRL1 + siENO1‐3, pcDNA3.1‐FGFRL1 + ENOblock or pcDNA3.1‐FGFRL1 + LY294002. *P < .05; **P < .01; ***P < .001

Increasing evidence has shown that ENO1 can function as an oncogenic protein by promoting cell proliferation, invasion and metastasis in many cancers.^17^, ^19^, ^20^, ^21^, ^22^, ^25^, ^26^ The present research described above also confirmed that ENO1 is highly expressed in chemoresistant SCLC cells. To examine whether ENO1 promotes chemoresistance of SCLC, we knocked down its expression in chemoresistant SCLC cells and then analysed drug resistance. The efficiency of siENO1 knockdown in H69AR cells was confirmed by qRT‐PCR and Western blot (Figure 5A). Accordingly, we chose siENO1‐2 and siENO1‐3 for the subsequent experiments. CCK8 assays showed that the IC50 values were significantly decreased after ENO1 knockdown in H69AR and H446DDP cells (Figure 5B). Moreover, an ENO1 inhibitor (ENOblock) gave the same results as the siRNA (Figure 5B). These results indicate that ENO1 indeed regulates chemoresistance of SCLC.

To explore whether ENO1 cooperates with FGFRL1 to promote chemoresistance of SCLC, we conducted CCK8 assays with down‐regulation or inhibition of ENO1 in FGFRL1‐overexpressing cells. The results showed significantly increased IC50 values in FGFRL1 overexpression cells compared with the empty vector controls, and knockdown or inhibition of ENO1 by siRNA or ENOblock in FGFRL1 overexpression cells could rescue the increase in IC50 values mediated by FGFRL1 up‐regulation (Figure 5C). These results suggest that FGFRL1 potentially mediates chemoresistance of SCLC via ENO1.

### 3.6 FGFRL1 regulates ENO1 expression and the PI3K/Akt pathway

**Figure 6:**
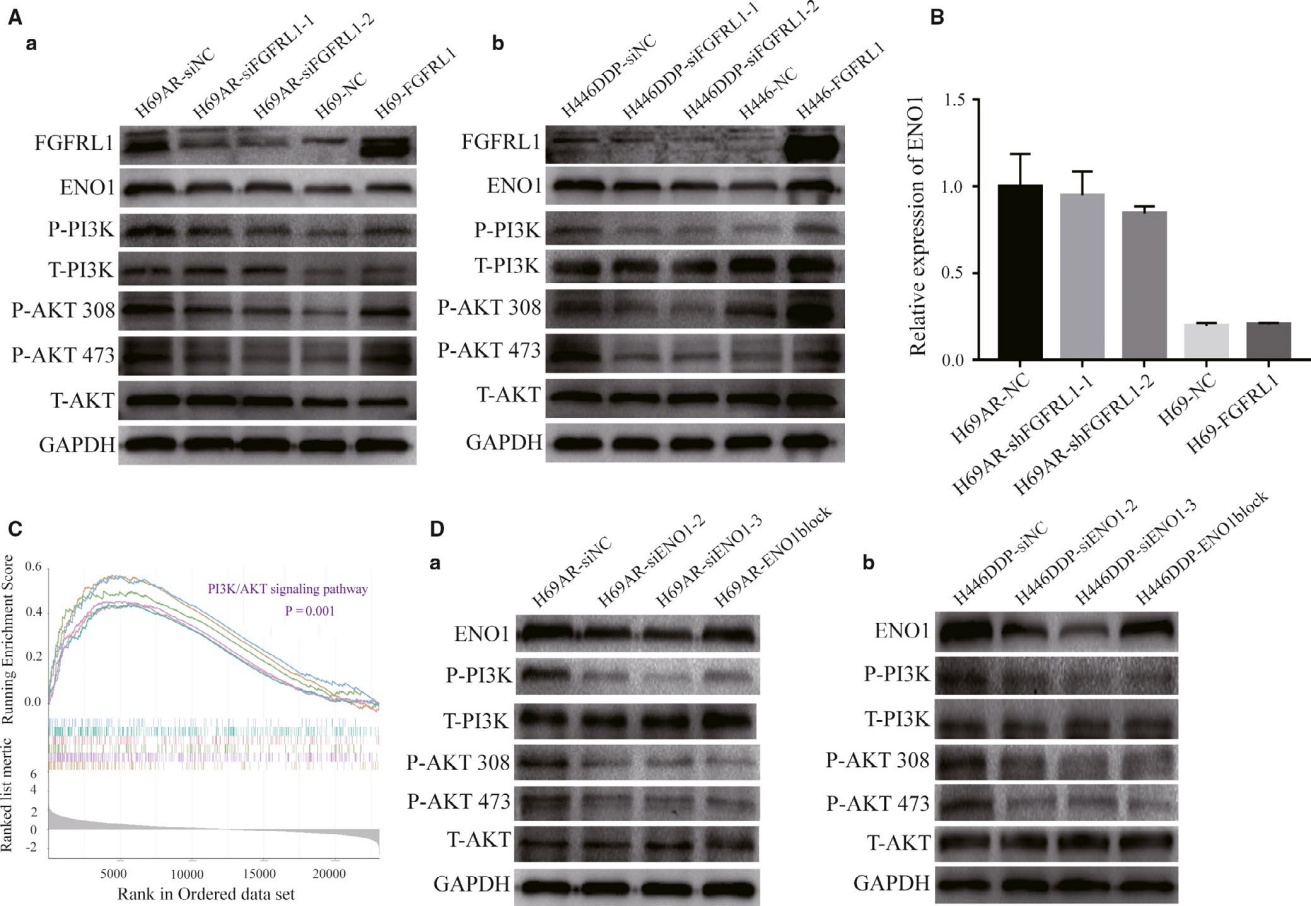
FGFRL1 regulates ENO1 expression and the PI3K/Akt pathway. A, Western blot analysis of FGFRL1, ENO1 and the phosphorylation of PI3K, and AKT in FGFRL1–down‐regulated or FGFRL1–up‐regulated SCLC cells. B, The expression of ENO1 was detected by qRT‐PCR in FGFRL1–down‐regulated or FGFRL1–up‐regulated SCLC cells. C, The GSEA plot showing the involvement of the PI3K/Akt signalling pathway. D, ENO1 expression and the phosphorylation levels of PI3K and AKT were assessed by Western blot in ENO1–down‐regulated or ENO1–inhibited cells

Given that ENO1 mediated the chemoresistance induced by FGFRL1 overexpression in SCLC, we wondered whether FGFRL1 could regulate the expression of ENO1 in SCLC cells. We found that the protein level of ENO1 was down‐regulated in the FGFRL1 knockdown cells and up‐regulated in the FGFRL1 overexpression cells (Figure 6A), but there was no significant change in transcription level (Figure 6B). It has been confirmed that ENO1 can regulate the PI3K/AKT pathway positively.^19^, ^21^, ^25^ According to the sequencing data, SCLC patients were equally separated into high, medium and low FGFRL1 expression groups.^27^ Differential analysis and enrichment analysis were performed in patients with high and low expression of FGFRL1. The GSEA plot shows that members of the PI3K/Akt signalling pathway were among the differentially expressed genes (Figure 6C). Therefore, we studied whether the PI3K/Akt signalling pathway is involved in FGFRL1‐mediated SCLC chemoresistance. As shown in Figure 6A, silencing of FGFRL1 reduced phosphorylation levels of PI3K and AKT (T308 and S473), and there is a similar decrease in knockdown or inhibition of ENO1 (Figure 6D). In the above three groups, the total levels of PI3K and AKT remained unchanged. In contrast, the phosphorylation levels of PI3K and AKT (T308 and S473) are significantly increased because of overexpression of FGFRL1 (Figure 6A). Treatment of FGFRL1 overexpression cells with LY294002 had a rescue effect on the IC50 value of SCLC as ENO1 knockdown or inhibition (Figure 5C). These results demonstrated that FGFRL1 mediates chemoresistance of SCLC by regulating ENO1 expression and its downstream signalling pathway.

## 4 DISCUSSION

Mice with FGFRL1 knockout are born normally but die after birth because of hypoplastic diaphragm at once.^28^ A patient was diagnosed with Antley‐Bixler syndrome caused by a frameshift mutation in the FGFRL1 gene on chromosome 4p16.^29^ Emerging evidence has demonstrated that FGFRL1 is related to tumour occurrence, development and metastasis, such as prostate cancer, gastric cancer, ovarian cancer and others.^11^, ^12^, ^13^, ^14^, ^15^, ^16^ But the mechanism of FGFRL1 in the drug resistance of SCLC is still unclear. In the present study, we first demonstrated that FGFRL1 expression is elevated in chemoresistant SCLC cells. We further showed that down‐regulation or overexpression of FGFRL1 could weaken or potentiate chemosensitivity, cell apoptosis and cell cycle arrest. As far as we know, our study is the first time to demonstrate the role of FGFRL1 in SCLC chemoresistance.

FGFRL1 is a member of the FGFR family. Although FGFRL1 is lack of the classical kinase domain, it still exerts great influence on signal pathways, perhaps through interactions with key signalling proteins. Silva et al^10^ showed that overexpression of FGFRL1 gives birth to activating ERK1/2 signalling by interacting with SHP1 in pancreatic beta cells. Zhuang et al^30^ found that FGFRL1 binds members of the Sprouty/Spred family to modulate the FGF signalling pathway during the morphogenesis of branch lung and renal epithelial tubes. To explore the mechanisms of FGFRL1 underlying chemoresistance of SCLC, we performed immunoprecipitation‐mass spectrometry analysis using FGFRL1‐specific antibody to screen for FGFRL1 binding partners in SCLC cells. Our research demonstrated that ENO1 showed high coverage with FGFRL1 and high expression in the chemoresistant SCLC cells. Then, we confirmed that FGFRL1 interacts with ENO1 in SCLC cells by reverse Co‐IP and immunofluorescence assays.

ENO1 is a glycolytic enzymes involved in certain key biological process in tumorigenesis, proliferation, migration and invasion of cancer.^23^ ENO1 catalyses the dehydration of 2‐PG to PEP during glycolysis^22^ and acts as a positive regulator of the PI3K/Akt pathway. Some studies have reported that lncRNAs or proteins can interact with ENO1 and affect its expression or activity in tumours. Chen et al^19^ demonstrated that WW domain‐binding protein 2 interacts with ENO1 and affects its expression. Yu et al^23^ showed that lncRNA‐6195 can inhibit the progression of HCC by binding to ENO1 and mitigating its biological activity. We demonstrated that ENO1 expression was increased in the chemoresistant SCLC cells, and down‐regulation of ENO1 resulted in increased cell sensitivity to chemotherapy drugs. In addition, we found that enhanced expression of FGFRL1 promoted ENO1 expression and its downstream PI3K/Akt pathway in SCLC cells, whereas decreased expression of FGFRL1 had the opposite effects. Zhan et al^24^ found that FBXW7, an E3 ligase, negatively regulates the expression of ENO1 by interacting with ENO1 and increasing its proteasomal degradation in colorectal cancer. Therefore, we hypothesize that FGFRL1 may compete with FBXW7 to bind ENO1, reducing the degradation of ENO1 in SCLC cells. More research is needed to confirm this hypothesis.

**Figure 7:**
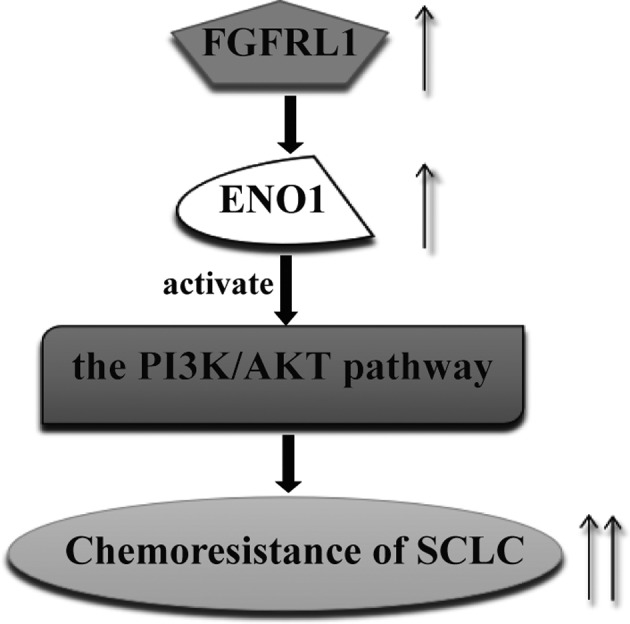
Schematic diagram showing that FGFRL1 modulates chemoresistance of SCLC through regulation of the ENO1‐PI3K/Akt signalling pathway by binding ENO1 in SCLC cells

In summary, our research indicated that FGFRL1 expression is associated with clinical stage and survival in SCLC patients. FGFRL1 could affect the chemosensitivity of SCLC cells in vitro and in vivo. Additionally, we revealed that FGFRL1 modulates chemoresistance of SCLC by regulating the ENO1‐PI3K/Akt pathway via combining to ENO1 in SCLC cells (Figure 7). These findings suggest that FGFRL1 may be a predictor and a potential therapeutic target against chemoresistance of SCLC.

## CONFLICT OF INTEREST

The authors declare no conflict of interest.

## AUTHOR CONTRIBUTIONS

S. Fang, L. Guo and R. Chen conceived and designed the experiments. R. Chen, D. Li and M. Zheng performed the experiments. R. Chen, T. Wei, W. Huang and B. Chen analysed the data. R. Chen, Q. Tong, Y. Wang, M. Li, Y. Zhu and W. Fang wrote the paper. All authors read and approved the final manuscript.

## FUNDING INFORMATION

Natural Science Foundation of Guangdong Province (2016A030310385); National Natural Science Foundation of China (81601986).
