# Supplementary material for: FGFRL1 affects chemoresistance of small‐cell lung cancer by modulating the PI3K/Akt pathway via ENO1
Source: J Cell Mol Med. 2020 Jan 19;24(3):2123–34. doi: 10.1111/jcmm.14763 (PMC7011138; doi:10.1111/jcmm.14763)
Supplement: Supplementary file 1 [file JCMM-24-2123-s001.docx]

**Supplementary materials**

Supplementary Legends and Figures

Figure S1. (A) (a) mRNA expression of ABCB1 and ABCG2 in two pairs of chemoresistant SCLC cell lines; (b and c) IC50 values of two drug-resistant cells were measured by CCK8 assays. (B) The expression of FGFRL1 was assessed in SCLC cell lines compared with the normal bronchial epithelial cell line (HBE) by qRT-PCR and western blot. (C) FGFRL1 inhibition by transfection of FGFRL1 siRNA in H69AR cells. (D) qRT-PCR and western blot were performed to evaluate the expression of FGFRL1 in the tumor xenografts. *, *P* < 0.05; **, *P* < 0.01; ***, *P* < 0.001.

Figure S2. (A and B) Cell apoptosis and cell cycle arrest were tested by flow cytometric analysis in FGFRL1-downregulated SCLC cells after CDDP exposure. (C and D) Flow cytometric analysis of cell apoptosis and cell cycle arrest induced by CDDP in FGFRL1-overexpressing SCLC cells.

Figure S3. (A and B) Cell apoptosis and cell cycle arrest were tested by flow cytometric analysis in FGFRL1-downregulated SCLC cells after VP16 exposure. (C and D) Flow cytometric analysis of cell apoptosis and cell cycle arrest induced by VP16 in FGFRL1-overexpressing SCLC cells.

Figure S1


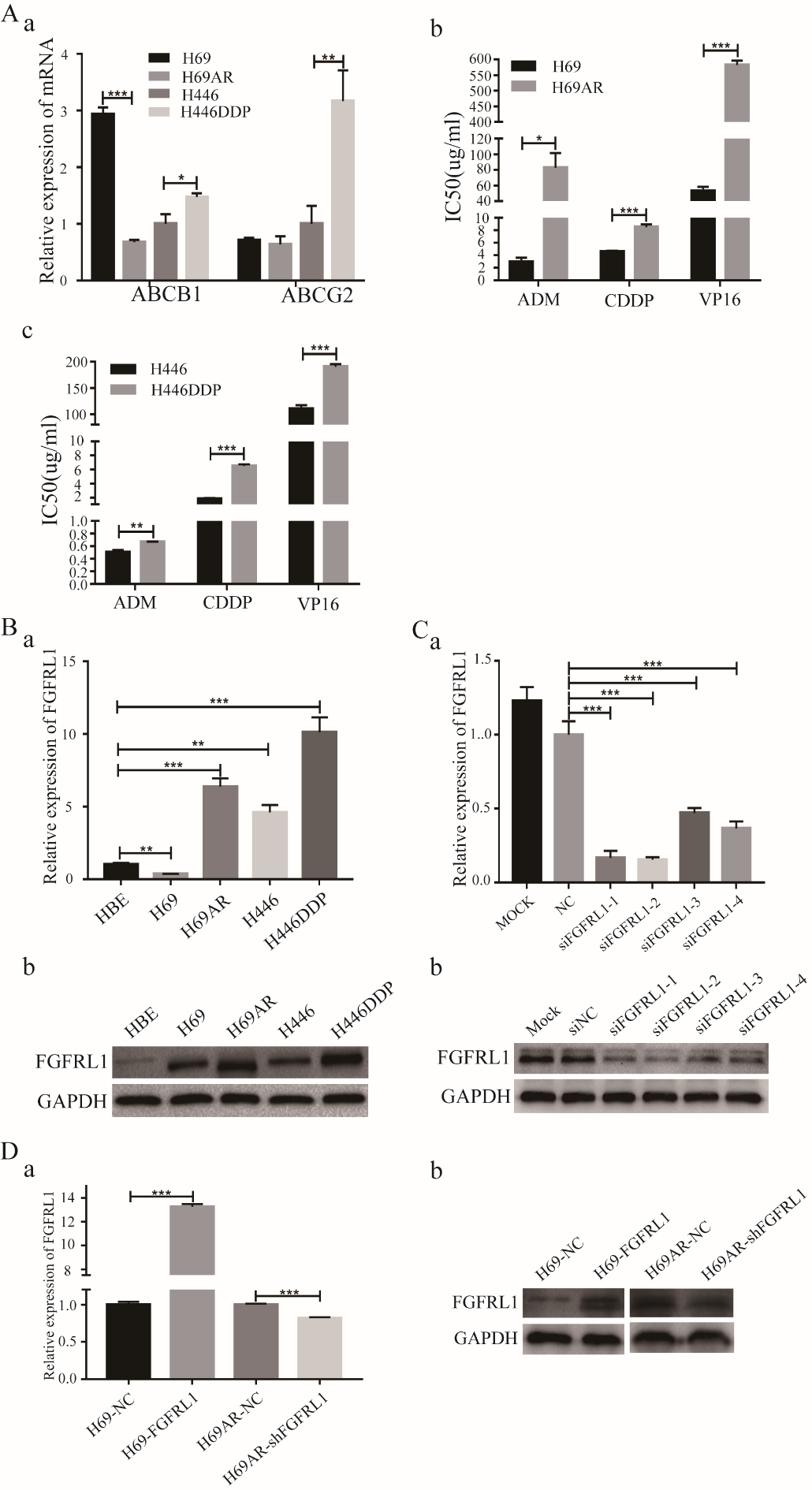


Figure S2


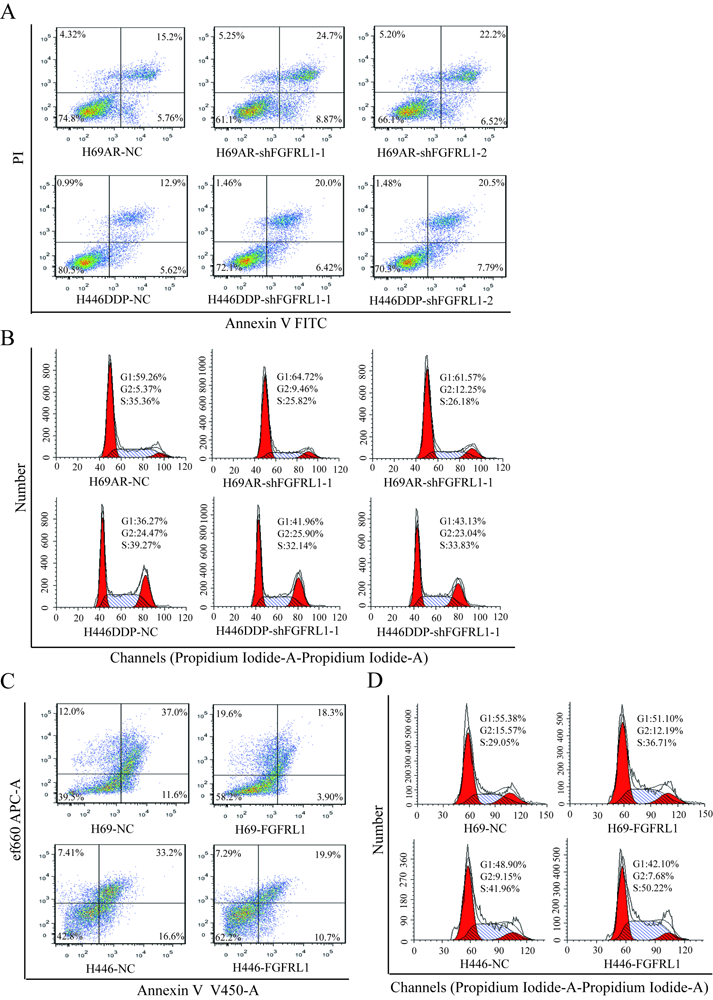


Figure S3


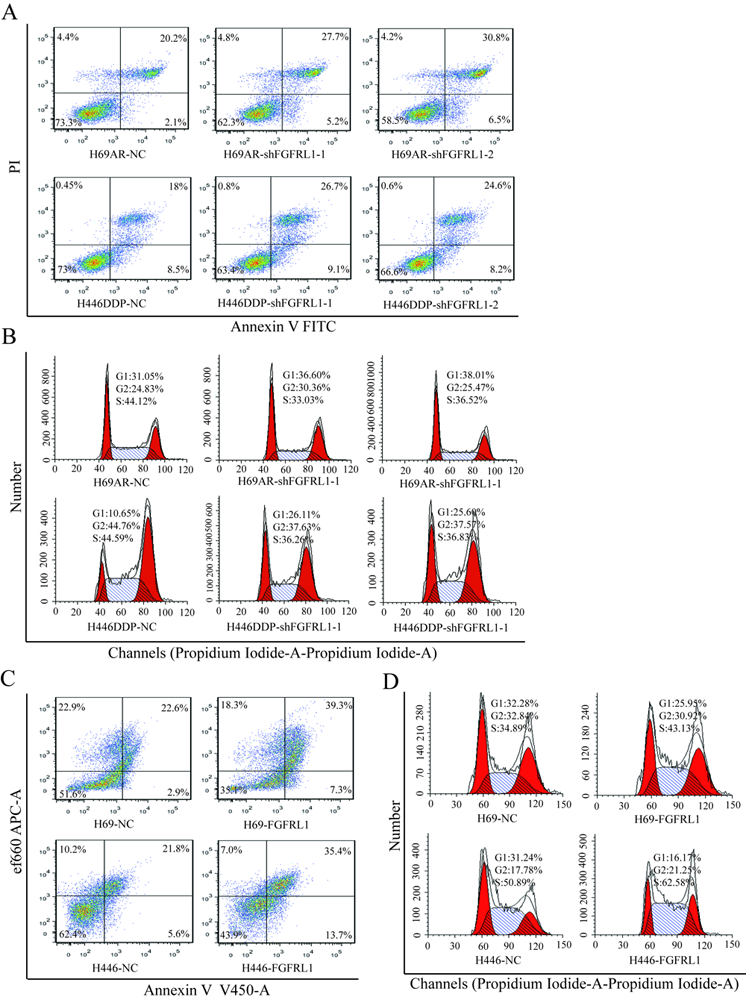


Table S1. qRT-PCR primers

| mRNA | Forward primer | Reverse primer |
| --- | --- | --- |
| FGFRL1 | CGCCATCAACGCCACCTACAAG | CGAAGTCCACCGTCGTGTTCAC |
| ENO1 | GTACCGCCACATCGCTGACTTG | AGCATGAGAACCGCCATTGATGAC |
| GAPDH | GCACCGTCAAGGCTGAGAAC | TGGTGAAGACGCCAGTGGA |

Table S2. Sequences of siRNA.

| RNAi | **Sense (5'-3')** | **Antisense (5'-3')** |
| --- | --- | --- |
| FGFRL1-Homo-497(FGFRL1-si1) | GCUGGAUGACAUUAGCCCATT | UGGGCUAAUGUCAUCCAGCTT |
| FGFRL1-Homo-748(FGFRL1-si2) | GGAAGAAGAAGUGGACACUTT | AGUGUCCACUUCUUCUUCCTT |
| FGFRL1-Homo-1048(FGFRL1-si3) | GCCAGAAGUUUGUGGUGCUTT | AGCACCACAAACUUCUGGCTT |
| FGFRL1-Homo-1841(FGFRL1-si4) | CUGGAUGCAUGUAUGCACATT | UGUGCAUACAUGCAUCCAGTT |
| ENO1-Homo-540（ENO1-si1） | CCAUGCCAGGGAGAUCUUUTT | AAAGAUCUCCCUGGCAUGGTT |
| ENO1-Homo-934（ENO1-si2） | GCUGGCAACUCUGAAGUCATT | UGACUUCAGAGUUGCCAGCTT |
| ENO1-Homo-1383（ENO1-si3） | CCCAGUGGUGUCUAUCGAATT | UUCGAUAGACACCACUGGGTT |

Table S3. Differentially expressed genes in H69AR and H69.

| **GeneSymbol** | **Fold change (H69AR/H69)** |
| --- | --- |
| FGFRL1 | 28.4665 |
| FSTL1 | 26.9913 |
| FBN2 | 14.8998 |
| FER1L3 | 12.7287 |
| FZD1 | 10.3590 |
| FOXC1 | 9.4415 |
| FN1 | 8.9388 |
| FCHO1 | 3.2172 |
| FGFR1OP2 | 2.7279 |
| FYCO1 | 2.7029 |
| FAT | 2.0050 |
| FOXA3 | 0.4182 |
| FBXO38 | 0.4087 |
| FYN | 0.3977 |
| FEN1 | 0.2670 |
| FGD3 | 0.2665 |
| FGF18 | 0.2645 |
| FER1L4 | 0.1788 |
| FGF14 | 0.1475 |
| FXYD6 | 0.0432 |
